# Osteomalacia in an HIV-infected man receiving rifabutin, a cytochrome P450 enzyme inducer: a case report

**DOI:** 10.1186/1476-0711-7-3

**Published:** 2008-01-28

**Authors:** Mark J Bolland, Andrew Grey, Anne M Horne, Mark G Thomas

**Affiliations:** 1 Department of Medicine, University of Auckland, Private Bag 92 019, Auckland 1020, New Zealand; 2 Department of Infectious Diseases, Auckland City Hospital, and Department of Molecular Medicine and Pathology, University of Auckland, Private Bag 92 019, Auckland 1020, New Zealand

## Abstract

**Introduction:**

People infected with human immunodeficiency virus are frequently treated with medications that can induce or inhibit cytochrome P450 enzymes.

**Case presentation:**

A 59 year old man treated with zidovudine, lamivudine, indinavir, and ritonavir for infection with human immunodeficiency virus volunteered to take part in a study of bone loss. He was found to have vitamin D insufficiency with secondary hyperparathyroidism and received vitamin D and calcium supplementation. He suffered a recurrence of infection with *Mycobacterium avium intracellulare *for which he received treatment with ciprofloxacin, rifabutin, and ethambutol. Subsequently, he developed worsening vitamin D deficiency with hypocalcaemia, secondary hyperparathyroidism and elevated markers of bone turnover culminating in an osteomalacic vertebral fracture. Correction of the vitamin D deficiency required 100,000 IU of cholecalciferol monthly.

Rifabutin is a cytochrome P450 inducer, and vitamin D and its metabolites are catabolised by cytochrome P450 enzymes. We therefore propose that treatment with rifabutin led to the induction of cytochrome P450 enzymes catabolising vitamin D, thereby causing vitamin D deficiency and osteomalacia. This process might be mediated through the steroid and xenobiotic receptor (SXR).

**Conclusion:**

Treatment with rifabutin induces the cytochrome P450 enzymes that metabolise vitamin D and patients treated with rifabutin might be at increased risk of vitamin D deficiency. In complex medication regimens involving agents that induce or inhibit cytochrome P450 enzmyes, consultation with a clinical pharmacist or pharmacologist may be helpful in predicting and/or preventing potentially harmful interactions.

## Introduction

Medications that induce or inhibit cytochrome P450 enzymes are commonly prescribed and therefore drug interactions mediated by changes in these enzymes are also common. People infected with human immunodeficiency virus (HIV) are frequently treated with complex medical regimens containing a number of medications that can induce or inhibit cytochrome P450 enzymes. We present a case of osteomalacia in an HIV-infected man that was likely caused by cytochrome P450-mediated alterations in vitamin D metabolism.

## Case presentation

A 59 year old Sri Lankan man volunteered to take part in a clinical study of bone loss in men infected with HIV. He had been diagnosed with HIV infection five years earlier. He also had a history of bronchiectasis and infection with *Mycobacterium avium intracellulare *(MAIC). He had been treated with zidovudine, lamivudine and indinavir for four years. Ritonavir 100 mg twice daily was added two years earlier. Ritonavir is a potent cytochrome P450 inhibitor that inhibits the metabolism of indinavir allowing it to be taken in smaller doses at less frequent intervals [1]. He also was taking clarithromycin and ethambutol for MAIC prophylaxis and cotrimoxazole for *Pneumocystis jiroveci *prophylaxis. His CD4 count was 41 cells/μL and viral load undetectable at time of study entry. Both parameters had been stable at these levels for more than 12 months prior to study entry.

**Figure 1:**
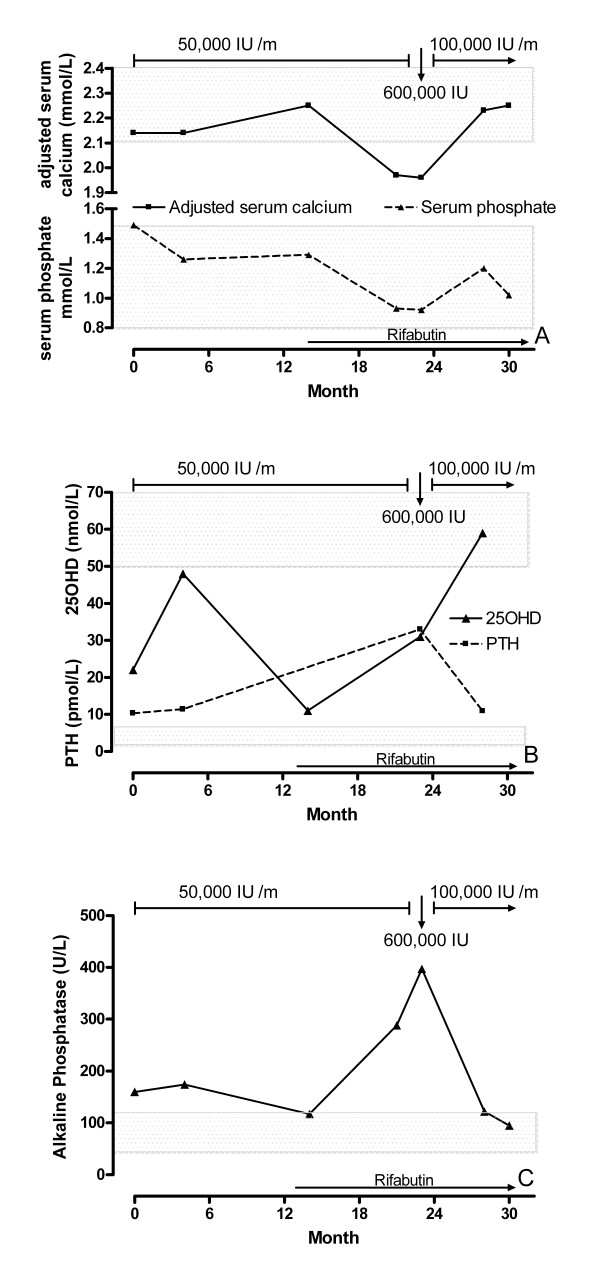
Panel A show the changes over time in adjusted serum calcium and serum phosphate, Panel B the changes in 25-hydroxyvitamin D (25OHD) and parathyroid hormone (PTH) and Panel C the changes in alkaline phosphatase. Monthly doses of cholecalciferol are indicated at the top of each panel. Treatment with rifabutin for infection with MAIC started at month 14. Reference ranges are indicated by the shaded boxes: adjusted serum calcium 2.10–2.55 mmol/L; phosphate 0.70–1.50 mmol/L; alkaline phosphatase 40–120 IU/L; parathyroid hormone 1.7–7.3 pmol/L.

Biochemical tests at the time of study entry and subsequently are shown in Figure 1. At the baseline visit he had vitamin D deficiency [25-hydroxyvitamin D (25OHD) <25 nmol/L] [2] with secondary hyperparathyroidism and a raised alkaline phosphatase (ALP). Liver and kidney function were normal. Bone mineral density (BMD) at the total hip was measured with a Lunar Expert dual energy x-ray absorptiometer and was 0.613 g/cm^2^. He subsequently received 400 mg calcium daily and 50,000 IU vitamin D (cholecalciferol) monthly. After four months his 25OHD level had increased although it was still below the recommended level of 50 nmol/L [2]. The secondary hyperparathyroidism and elevated ALP persisted.

After 14 months he developed recurrence of MAIC infection, and treatment was commenced with ciprofloxacin, rifabutin, and ethambutol. Blood tests at this time showed normalisation of ALP but a low 25OHD level (Figure 1) despite reported full compliance with medication. Over the next nine months his blood tests showed worsening vitamin D deficiency, development of hypocalcaemia, secondary hyperparathyroidism, and progressive elevation of ALP. After 21 months, he developed lower back pain and generalised myalgia. Magnetic resonance imaging showed a fracture of the endplate of L1. His symptoms and biochemical tests were consistent with osteomalacia although confirmatory bone biopsy was not performed. BMD at the total hip decreased by 17% between 12 and 23 months.

When the diagnosis of osteomalacia was recognised at 23 months, he was treated with a loading dose of 600,000 IU of cholecalciferol and thereafter 100,000 IU per month, and 1 g of calcium daily. This produced a rapid normalisation of his serum calcium, phosphate, ALP and 25OHD, and resolution of the musculoskeletal symptoms, although serum parathyroid hormone remained mildly elevated.

## Discussion

Vitamin D insufficiency (25OHD <50 nmol/L) [2] is common in adults, especially in the elderly, and causes myopathy, osteopenia, secondary hyperparathyroidism, and osteomalacia [3,4]. The prevalence of osteomalacia in adults is unknown but is thought to be rare outside high risk groups such as the frail elderly [4].

**Figure 2:**
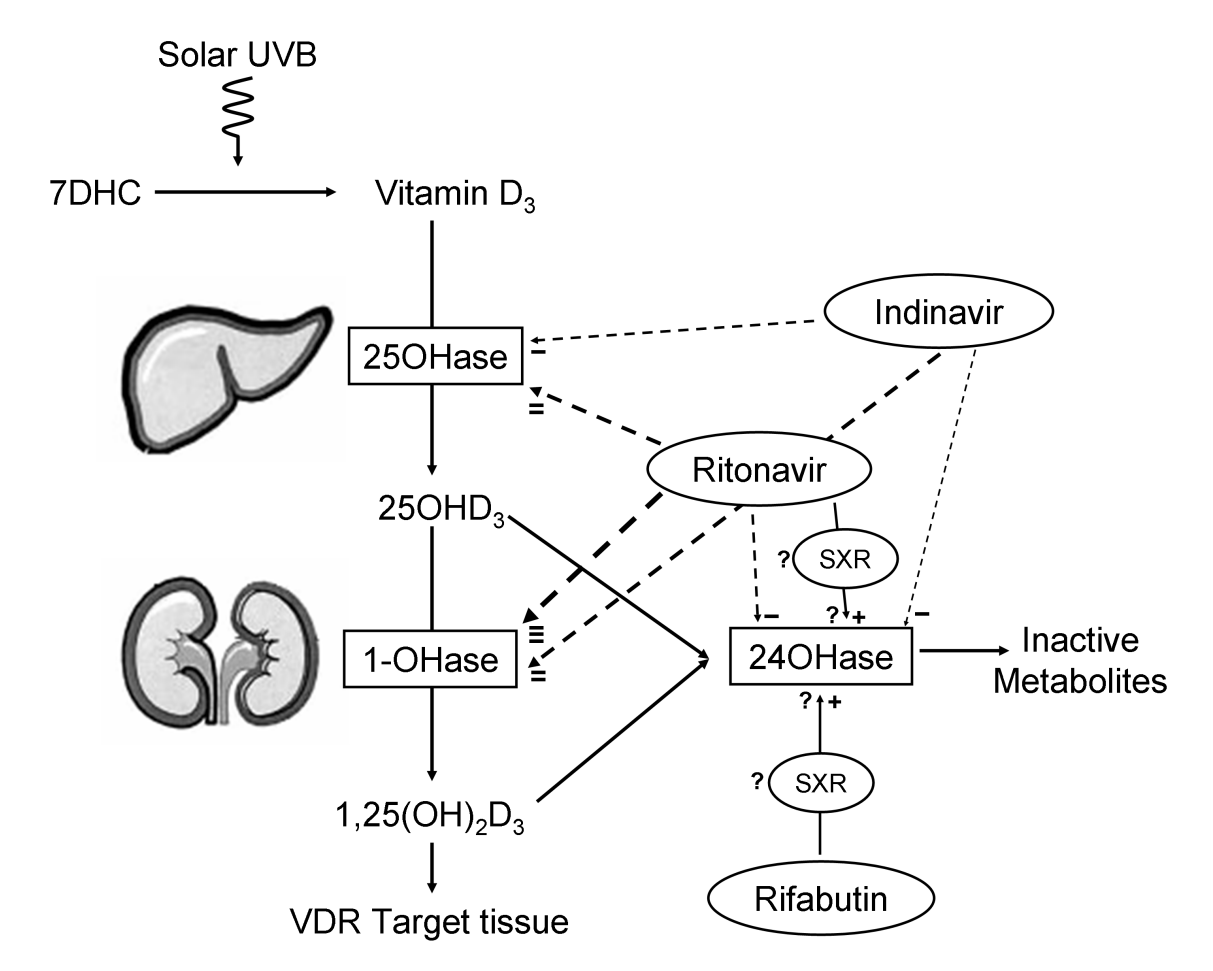
Schematic diagram of vitamin D metabolism and cytochrome P450 mediated drug effects. Cytochrome P450 enzyme inhibition is indicated by broken lines and stimulation by solid lines. The strength of enzyme inhibition or stimulation is indicated by the width of the lines and the number of terminal plus or minus signs. As the differential effects of rifabutin on the vitamin D hydroxylase enzymes have not been reported, our prediction of the action of rifabutin is shown, which might be mediated by SXR. Ritonavir also can bind to SXR which might induce 24-hydroxylase activity, but *in vitro *ritonavir appears to inhibit rather than stimulate 24-hydroxylase. Abbreviations: 7DHC 7-dehydrocholesterol; 25OHase 25-hydroxylase; 25OHD_3 _25-hydroxyvitamin D3; 1OHase 1α-hydroxylase; 1,25(OH)_2_D_3 _1,25-dihydroxyvitamin D_3_; VDR vitamin D receptor; 24OHase 24-hydroxylase; SXR steroid and xenobiotic receptor.

The major source of vitamin D is from photolysis of steroid precursors in the skin, with only a small dietary contribution. Vitamin D is then hydroxylated in the liver to 25OHD by a cytochrome P450 enzyme called 25-hydroxylase. 25OHD is further hydroxylated in the kidney by a cytochrome p450 enzyme, 1α-hydroxylase, to 1,25-dihydroxyvitamin D [1,25(OH)_2_D], the most active form of vitamin D. Other cytochrome P450 enzymes such as 24-hydroxylase metabolise the hydroxylated forms of vitamin D to inactive metabolites which are excreted in the bile. Each of these cytochrome P450 enzymes can be stimulated or inhibited by medication (Figure 2).

Our patient was treated with a number of medications that influence cytochrome P450 enzymes. At study entry he was treated with ritonavir, indinavir and clarithromycin – all cytochrome P450 inhibitors – and later he received rifabutin, a cytochrome P450 stimulator. Ritonavir is a potent cytochrome P450 inhibitor that has differential effects on vitamin D hydroxylase enzymes. *In vitro*, it is a potent inhibitor of both 1α-hydroxylase and 25-hydroxylase and a weak inhibitor of 24-hydroxylase. Thus it would be predicted *in vivo*, to lower levels of both 25OHD and 1,25(OH)_2_D [5]. Indinavir is a weaker cytochrome P450 inhibitor and would be predicted to have similar but lesser effects [5]. The effects of ritonavir and indinavir on vitamin D metabolism are expected to be similar to those described with isoniazid, another cytochrome P450 inhibitor [6]. Clarithromycin is a cytochrome P450 inhibitor that appears to be a less potent inhibitor than erythromycin [7]. Neither clarithromycin nor erythromycin has been reported to affect vitamin D metabolism *in vivo*, but effects similar to those described with ritonavir and isoniazid would be predicted.

Rifabutin is a cytochrome P450 inducer that is structurally similar to rifampicin, but a less potent enzyme stimulator [8]. The effects of rifabutin on vitamin D metabolism have not been reported but a two week course of rifampicin was reported to lower 25OHD levels by 70% in eight young men without altering levels of 1,25(OH)_2_D or parathyroid hormone [9]. The effects of rifampicin on the individual vitamin D hydroxylases have not been reported. While osteomalacia is well known to occur with the use of anticonvulsants, which are potent inducers of catabolic vitamin D hydroxylases, osteomalacia does not appear to be common with use of rifampicin and isoniazid [10].

Emerging evidence suggests that the orphan nuclear receptor, steroid and xenobiotic receptor (SXR), may have a key role in the development of drug-induced osteomalacia [11,12]. Drugs such as rifampicin bind to SXR, which stimulates 24-hydroxylase activity (Figure 2) thereby causing increased clearance of vitamin D metabolites, vitamin D deficiency and osteomalacia. Because of its structural similarities to rifampicin, rifabutin may also bind to SXR, although such an interaction has not yet been described. Protease inhibitors can also bind to SXR but have variable ability to activate its target genes, with ritonavir but not indinavir able to activate SXR-regulated pathways [13].

Our patient had vitamin D insufficiency at entry to the study, a relatively common finding in immigrants or native-born people with darkly pigmented skin in New Zealand [14]. Despite vitamin D replacement in doses that would usually promote adequate levels of 25OHD, he did not become vitamin D sufficient. This may have been due to ritonavir, indinavir and clarithromycin mediated inhibition of the cytochrome P450 enzymes that regulate generation of biologically active vitamin D. Poor compliance may have also contributed, although the patient reported taking all his medication. Another possibility is that conversion of 25OHD to 1,25(OH)_2_D by activated macrophages in MAIC-related granulomata may have lowered 25OHD levels. Subsequently, the addition of rifabutin was followed by the rapid development of osteomalacia, presumably due to cytochrome P450 enzyme induction that favoured catabolism of 25OHD. Treatment with doses of cholecalciferol, equivalent to 3,300 IU/day, was required to achieve levels of 25OHD that enabled normalisation of serum calcium, phosphate and ALP.

## Conclusion

In conclusion, this case demonstrates the importance of medication-induced alterations in cytochrome P450 enzymes involved in vitamin D metabolism. Patients with HIV infection often are at risk of opportunistic infections, or reactivation of tuberculosis infection. In these situations complex treatment regimens are used which often involve multiple agents that are capable of affecting cytochrome P450 enzymes. Clinicians need to be aware of potential cytochrome P450 interactions, and in complex medication regimens, consultation with a clinical pharmacist or pharmacologist may be helpful in predicting and/or preventing potentially harmful interactions.

## Competing interests

The author(s) declare that they have no competing interests.

## Authors' contributions

All authors were involved in the care of the patient. MB drafted the manuscript. AG, AH, MT critically reviewed and improved the manuscript. All authors read and approved the final manuscript.

## Consent

Written informed consent was obtained from the patient for participation in this research. A copy of the written consent is available for review by the Editor-in-Chief of this journal.
